# Zinc transporter mutations linked to acrodermatitis enteropathica disrupt function and cause mistrafficking

**DOI:** 10.1016/j.jbc.2021.100269

**Published:** 2021-01-08

**Authors:** Eziz Kuliyev, Chi Zhang, Dexin Sui, Jian Hu

**Affiliations:** 1 Department of Chemistry, Michigan State University, East Lansing, Michigan, USA; 2 Department of Biochemistry and Molecular Biology, Michigan State University, East Lansing, Michigan, USA

**Keywords:** acrodermatitis enteropathica, ZIP4, zinc transporter, disease-causing mutation, extracellular domain, mistrafficking, misfolding, AE, acrodermatitis enteropathica, CD, circular dichroism, CF, cystic fibrosis, CFTR, cystic fibrosis transmembrane conductance regulator, CLSM, confocal laser scanning microscope, DMEM, Dulbecco’s modified eagle medium, ECD, extracellular domain, FBS, fetal bovine serum, HRD, helix-rich domain, LOF, loss-of-function, NBD1, nucleotide binding domain 1, PCC, Pearson's correlation coefficient, SCD-EDS, spondylocheirodysplastic form of Ehlers–Danlos syndrome, SNP, single-nucleotide polymorphism, TMD, transmembrane domain, ZIP, Zrt-/Irt-like protein

## Abstract

ZIP4 is a representative member of the Zrt-/Irt-like protein (ZIP) transporter family and responsible for zinc uptake from diet. Loss-of-function mutations of human ZIP4 (hZIP4) drastically reduce zinc absorption, causing a life-threatening autosomal recessive disorder, acrodermatitis enteropathica (AE). These mutations occur not only in the conserved transmembrane zinc transport machinery, but also in the extracellular domain (ECD) of hZIP4, which is only present in a fraction of mammalian ZIPs. How these AE-causing ECD mutations lead to ZIP4 malfunction has not be fully clarified. In this work, we characterized all seven confirmed AE-causing missense mutations in hZIP4-ECD and found that the variants exhibited completely abolished zinc transport activity in a cell-based transport assay. Although the variants were able to be expressed in HEK293T cells, they failed to traffic to the cell surface and were largely retained in the ER with immature glycosylation. When the corresponding mutations were introduced in the ECD of ZIP4 from *Pteropus Alecto*, a close homolog of hZIP4, the variants exhibited structural defects or reduced thermal stability, which likely accounts for intracellular mistrafficking of the AE-associated variants and as such a total loss of zinc uptake activity. This work provides a molecular pathogenic mechanism for AE.

Zinc is an essential micronutrient for any living organism. As the second most abundant transition metal element in humans after iron, zinc is required for function of over 2000 transcription factors and activity of approximately 300 enzymes (1). It has been estimated that zinc ions bind to nearly 3000 proteins, which account for ∼10% of the proteins encoded in human genome (2). Studies have shown that zinc deficiency may affect many biological processes, causing growth retardation, immune dysfunction, diarrhea, delayed sexual maturation, and skin lesions (3). Zinc deficiency is usually caused by inadequate zinc supply in foods or acquired diseases either reducing zinc uptake or increasing zinc loss (4). In a rare case, inherited zinc deficiency, which is called acrodermatitis enteropathica (AE), is caused by loss-of-function (LOF) mutations of ZIP4 (5, 6), the exclusive high-affinity zinc transporter in gastrointestinal system responsible for zinc uptake from regular diet (7, 8, 9, 10). AE is fatal without treatment, but lifelong high-dose zinc supplementation on daily basis can effectively alleviate the symptoms (11), implying the presence of secondary low-affinity zinc absorption mechanism(s).

ZIP4 is a representative member of the Zrt-/Irt-like protein (ZIP) family (solute carrier 39A, SLC39A) consisting of divalent transition metal transporters ubiquitous in all the kingdoms of life (12, 13, 14, 15, 16, 17, 18). ZIP4 is specifically expressed on the apical side of enterocytes in the small intestine and also in the kidney where it is believed to be involved in zinc reabsorption from urine (18). Topologically, ZIP4 has a transmembrane domain (TMD), which is generally conserved in the entire ZIP family, and a large extracellular domain (ECD), which is only present in a fraction of the family members (19, 20). The AE-causing mutations are evenly distributed along the 12 exons of *zip4* gene without showing hotspot (21, 22). As a result, half of the missense AE-causing mutations are mapped in the ECD and the other half in the TMD where the zinc transport machinery is located. A previous work has investigated several corresponding mutations in the TMD of mouse ZIP4 (mZIP4) but only one mutation in the ECD (equivalent to the P200L mutation in human ZIP4, hZIP4) was studied in the same report (7). The P200L variant of hZIP4 was also characterized in a recent report (23). So far, it is still unclear about how the other AE-causing mutations in the ECD affect hZIP4 function and the corresponding molecular mechanisms are also unknown.

The crystal structure of the ECD of ZIP4 from *Pteropus Alecto* (black fruit bat, pZIP4-ECD), which shares 68% sequence identity with hZIP4-ECD, provides a structural framework to deduce structural impacts of the AE-causing mutations on this accessory domain required for optimal zinc transport (19). In this work, we functionally characterized all the seven confirmed AE-associated variants in a human cell line (HEK293T) and biophysically studied the purified variants of pZIP4-ECD harboring the corresponding mutations. We found that all the variants showed little zinc transport activity in the cell-based transport assay. Although the variants were expressed, the cell surface expression was barely detectable. The variants were found to be immaturely glycosylated and aberrantly retained in the ER. For the purified pZIP4-ECD variants, biophysical studies revealed that the mutations caused structural defects or reduced thermal stability, providing a possible causative explanation for ZIP4 mistrafficking and dysfunction.

## Results

### The AE-causing mutations in the ECD of hZIP4 led to total loss of zinc transport activity

**Figure 1:**
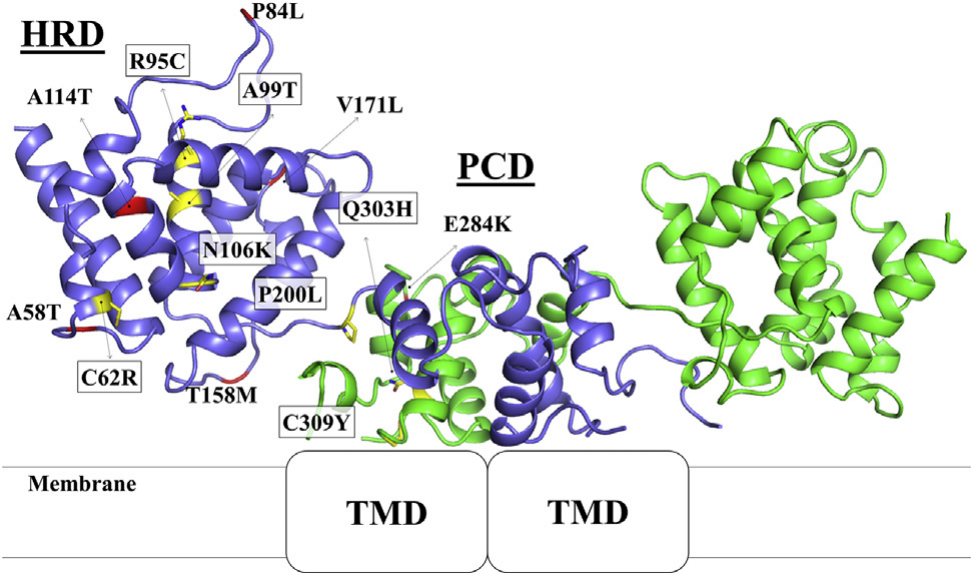
**Mapping of the AE-causing mutations and the SNPs on the structural model of hZIP4-ECD dimer.** Seven residues subjected to AE-causing mutations (highlighted in *boxes*) are in *yellow* and stick mode with four in the HRD domain, two in the PCD domain, and one (P200L) in the linker connecting the two subdomains, whereas the residues at which SNPs occur are in *red*. Additional three SNPs not shown in the model are either in the highly disordered his-rich loop (R251W) or in the signal peptide (V2A and E10A).

**Figure 2:**
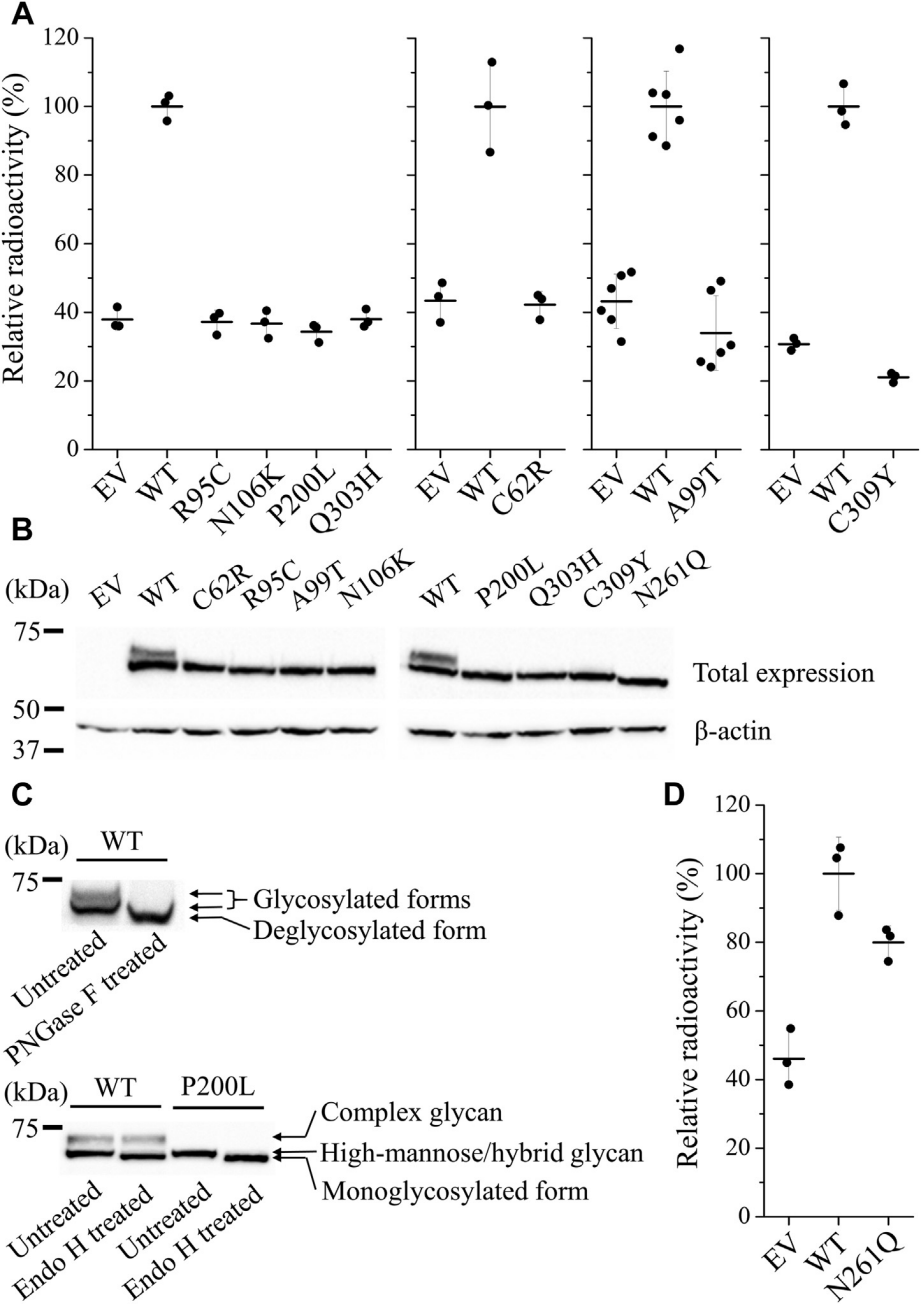
**Expression and functional characterization of hZIP4 and the variants.***A*, cell-based zinc transport assay of the AE-associated variants with 10 μM Zn^2+^ in the medium. The data points of one representative experiment out of 2–6 independent experiments are shown as *black dots*, and the means and the standard deviations are indicated by *bars*. *B*, total expression hZIP4 and the variants detected by western blot using anti-HA antibody. β-actin was used as loading control. *C*, processing of hZIP4 by PNGase F and Endo H glycosidases. *D*, zinc transport assay of the N261Q variant. Data are expressed as average ± standard deviation (n = 3).

A total of 17 missense mutations in hZIP4-ECD have been documented, of which seven are confirmed AE-causing mutations and the others are benign single-nucleotide polymorphisms (SNPs), according to the MASTERMIND database (https://mastermind.genomenon.com/) and an early review (21). Mapping these residues on the hZIP4-ECD structural model (generated using SWISS-MODEL (24)) shows that the residues subjected to AE-causing mutations (C62R, R95C, A99T, N106K, P200L, Q303H, and C309Y) are all in the structured regions, whereas most of the residues where the SNPs occur are in loops or disordered regions (Fig. 1). In this work, we characterized all the seven AE-causing mutations, including four homozygous mutations (C62R, P200L, Q303H, and C309Y) (5, 6, 25, 26) and three heterozygous mutations (R95C, A99T, and N106K) (6, 26, 27), and studied their impacts on hZIP4 function. The hZIP4 variants with a C-terminal HA tag were transiently expressed in HEK293T cells and applied to the cell-based zinc transport assay (6, 7, 28, 29). As shown in Figure 2*A*, in sharp contrast to the wild-type hZIP4, none of the cells expressing any variants uptook more zinc than the blank group in which the cells were transfected with an empty vector, which means that the variants have no detectable zinc transport activity with 10 μM of Zn^2+^ in culture media. For the wild-type hZIP4, the previous studies have shown that zinc transport activity reaches plateau at 10 μM of Zn^2+^ under the same condition (28, 29). To examine whether any activity can be detected at higher zinc concentration, the P200L variant was tested at various zinc concentrations up to 50 μM, but the results only confirmed no detectable activity (Fig. S1).

### The AE-associated variants were immaturely glycosylated

The previous study on mZIP4 has shown that the AE-causing mutations in the TMD led to significantly reduced expression levels, likely due to accelerated degradation (7). To examine whether the AE-causing mutations in the ECD have similar effects, expression of the variants was detected in the whole-cell samples using an anti-HA antibody in western blot. As shown in Figure 2*B*, all the variants were expressed, but the wild-type hZIP4 has two discrete bands at approximately 75 kDa, whereas the AE-associated variants have only one band corresponding to the lower band of the wild-type protein. When treated with PNGase F, an enzyme cleaving the glycosidic bond directly linked with the asparagine residue, the two bands of the wild-type protein merged into a single one with a smaller apparent molecular weight, indicating that the expressed hZIP4 harbors two distinct glycans (7). When treated with Endo H, which is a glycosidase selectively hydrolyzing high-mannose or some hybrid glycans, the lower band was processed, whereas the upper band was resistant (Fig. 2*C*), indicating that the species in the upper band has a complex glycan, whereas the lower band represents a species with immature glycosylation. In contrast, the P200L variant was completely processed by Endo H, and the resulting species is of the same apparent molecular weight as the one corresponding to the lower band of the processed wild-type protein. Thus, although the total expression levels of the variants were not drastically affected by the mutations in the ECD, defects in glycosylation were observed for all the AE-associated variants.

### N-glycosylation is not required for hZIP4 activity

Proteins expressed at cell surface are often modified by *N*-glycosylation, which may play a key role in protein function (30). As the AE-associated variants losing zinc transport activity are concomitant with defects in glycosylation, we asked whether the lack in glycosylation is responsible for activity loss. Using the NetNGlyc server (http://www.cbs.dtu.dk/services/NetNGlyc/), we identified a potential glycosylation site at N261 in an “NxS/T” motif. We then generated the N261Q variant and expressed it in HEK293T cells. In western blot, the N261Q variant showed a single band with an apparent molecular weight smaller than the lower band of the wild-type protein (Fig. 2*B*), confirming that N261 is indeed the only *N*-glycosylation site in hZIP4. Importantly, zinc transport assay of the N261Q variant only showed a modest reduction of transport activity (Fig. 2*D*), indicating that glycosylation at N261 is not pivotal for zinc transport and therefore lack of glycosylation of the AE-associated variants does not account for loss of zinc transport activity.

### The AE-associated variants had significantly diminished cell surface expression

**Figure 3:**
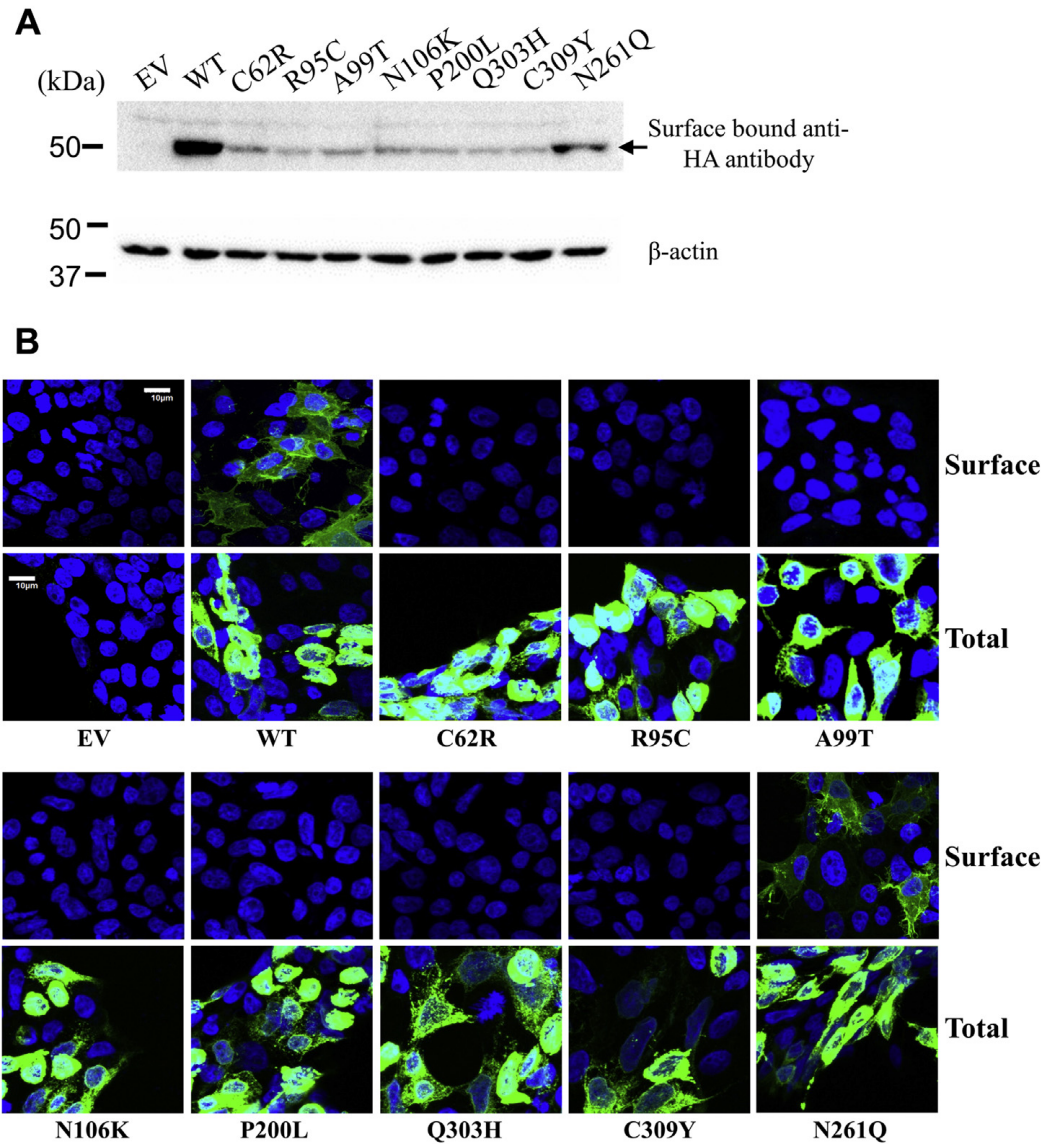
**Cell surface expression of hZIP4 and the AE-associated variants.***A*, surface-bound anti-HA antibody for detection of surface expression of hZIP4-HA and the variants. β-actin was used as the loading control. The result of one representative experiment out of three independent experiments is shown. *B*, detection of expression of hZIP4 and its variants. FITC-labeled anti-HA antibody was used to stain HA tagged hZIP4 in nonpermeabilized cells (surface expression, upper row) or permeabilized cells (total expression, lower row). The scale bar = 10 μm.

As the glycans are added to the client membrane proteins in the ER, processed in the ER and then in the Golgi in a stepwise manner (31), immature glycosylation is an indicator of defect in intracellular trafficking. To examine potential mislocalization, we tested cell surface expression levels of the AE-associated variants by applying the anti-HA antibody to the nonpermeabilized cells fixed with formaldehyde (7, 19, 32, 33). After extensive wash, nonspecific bound antibody was removed, leaving those specifically bound with the C-terminal HA tag at cell surface. As shown in Figure 3*A*, all the AE-associated variants had substantially reduced cell surface levels when compared with the wild-type hZIP4 in western blot. Consistent with the zinc transport data, the N261Q variant missing the *N*-linked glycan had significantly higher level of cell surface expression than the variants linked with disease, indicating that the *N*-glycosylation at N261 is not a key factor for hZIP4 surface expression. We then applied immunofluorescence imaging to locate the AE-associated variants expressed in cells. To do that, the cells transiently expressing the wild type or the variants were fixed and permeabilized with formaldehyde and TX-100, followed by staining with an FITC-labeled anti-HA antibody. The samples were then checked under confocal laser scanning microscope (CLSM). Consistent with the western blot shown in Figure 2*B*, the wild type and the variants were expressed in HEK293T cells at comparable levels (Fig. 3*B*). To detect the transporters expressed at cell surface, the cells were fixed with formaldehyde and treated with the same anti-HA antibody, followed by extensive washing and checking under CLSM. In Figure 3*B*, fluorescence signals at cell surface can be convincingly detected for the wild-type hZIP4 and the N261Q variant, whereas the signals of the AE-associated variants imaged under the same conditions were not detectable. Collectively, our data strongly indicate that the cell surface expression levels of the AE-associated variants were largely suppressed, which is likely responsible for loss of zinc transport activity.

### The AE-associated variants were retained in the ER

**Figure 4:**
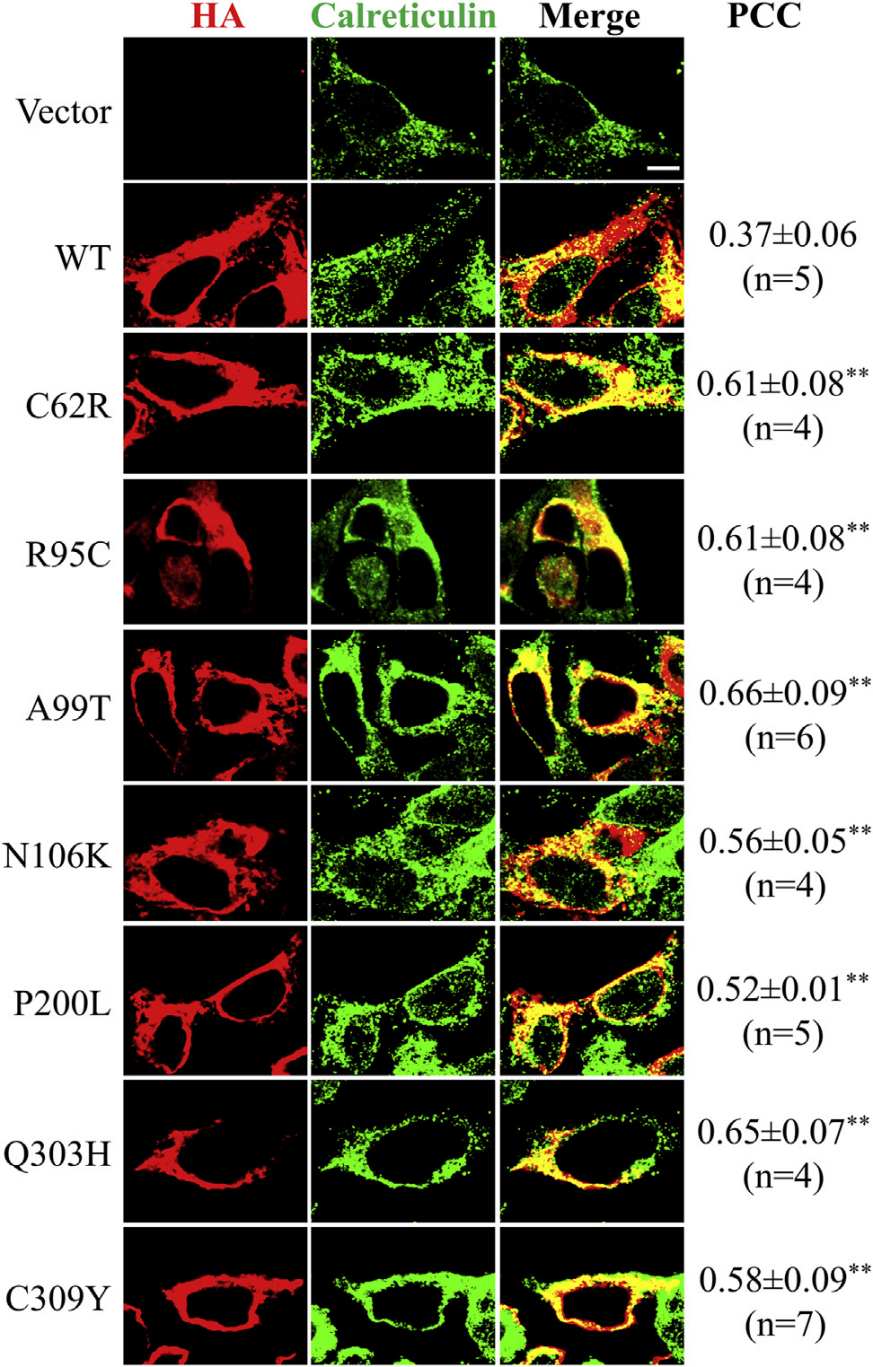
**Colocalization of hZIP4 with the ER marker calreticulin.** Representative confocal images (100×) of HEK293T cells transiently expressing hZIP4-HA or the variants are shown. hZIP4 and the variants were detected with an anti-HA tag antibody and an Alexa-568 goat anti-mouse antibody (*red*). Calreticulin was detected with an anti-calreticulin antibody and an Alexa-488 anti-rabbit antibody (*green*). Scale bar = 5 μm. Several dual-color images (n = 4–7) were taken and subjected to colocalization analysis using Image J equipped with the JACoP plugins. PCC was calculated for each image and data are expressed as average ± standard deviation for each construct. Statistical analysis was conducted to examine the significant difference of PCC between hZIP4 and the variants. ∗∗*p* < 0.01.

Since all the AE-associated variants were expressed but not presented at cell surface, we then asked where the variants are located in cells. Endo H treatment experiment suggests that the variants are likely trapped in the ER (Fig. 2*C*). To test this, we examined colocalization of hZIP4 and its variants with an ER-resident protein, calreticulin, and quantified the results using Pearson's correlation coefficient (PCC). As shown in Figure 4, the staining of the wild-type hZIP4-HA (red) was partially superimposed with those of calreticulin (green), resulting in a yellow/orange color. The calculated PCC (0.37 ± 0.06) indicates that a significant portion of hZIP4 expressed in HEK293T cells were retained in the ER, which is consistent with the results of the Endo H treatment, which showed that more than half of the expressed hZIP4 were not maturely glycosylated. For all the AE-associated variants, more overlapped signals were detected and the PCCs (0.52–0.66) are significantly higher than that of the wild-type protein (*p* < 0.01) (Fig. 4), indicating that these variants were retained in the ER to a greater extent than the wild-type protein.

### The AE-causing mutations led to structural defects or reduced thermal stability of pZIP4-ECD

Next, we attempted to clarify the molecular basis of the variants’ mistrafficking. Given the difficulty of obtaining adequate amount of purified full-length hZIP4 or hZIP4-ECD for biophysical characterization, we turned to the isolated pZIP4-ECD, which we have previously crystallized and biochemically characterized (19, 28). Given the high sequence identity, we believe that the results obtained from the study of purified pZIP4-ECD would help to understand how the disease-causing mutations affect the biophysical properties of the human counterpart. We introduced mutations in pZIP4-ECD on the residues topologically equivalent to those in hZIP4 (C64R, R87C, A91T, D98K, P193L, Q299H, and C305Y). The variants were expressed and purified in the same way as the wild-type protein, but the final yields were lower. Particularly, the yields of two variants (D98K and C305Y) were too low to allow for further characterization. Therefore, we focused on the other five variants in later study.

**Figure 5:**
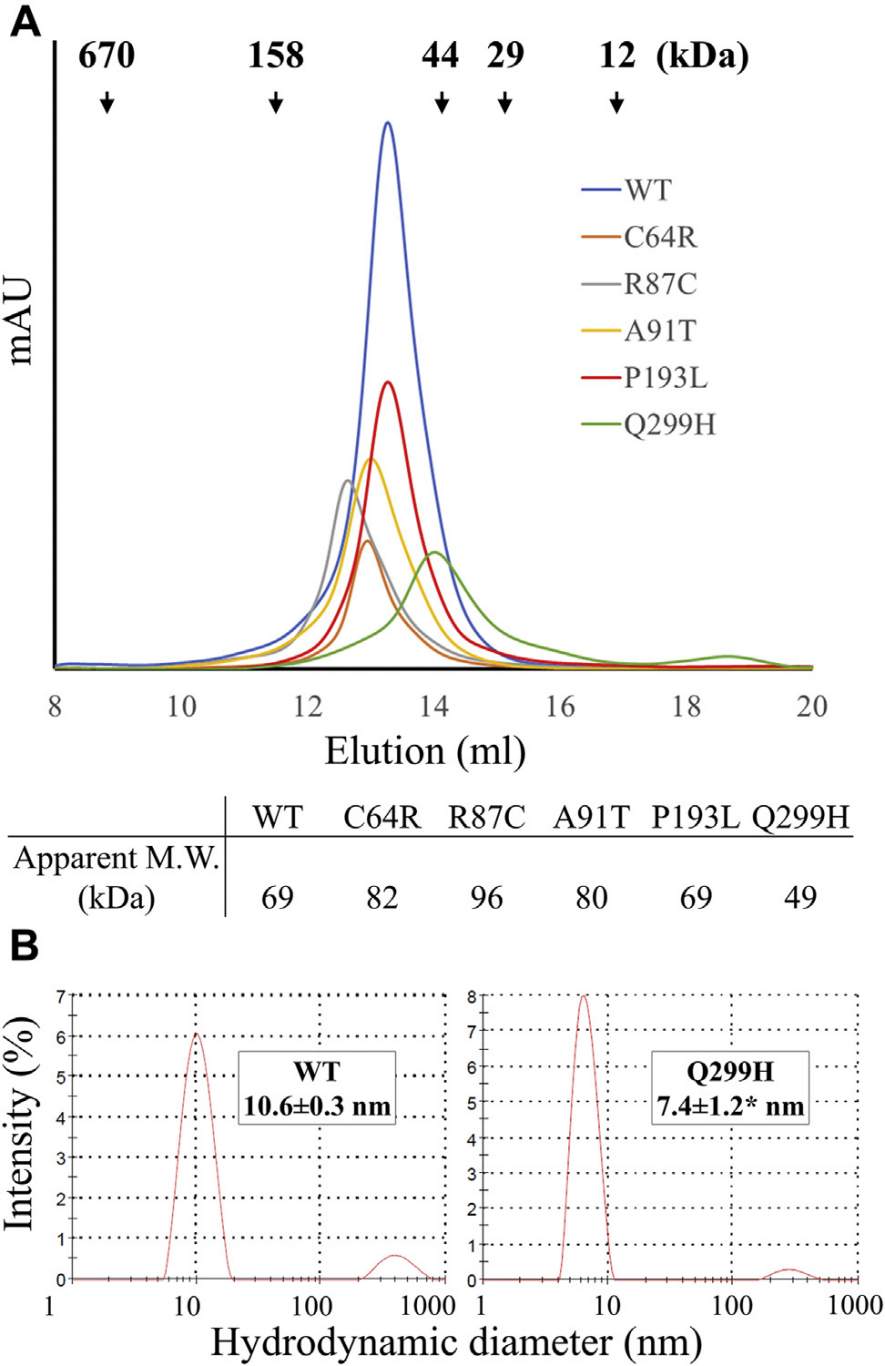
**Purification of pZIP4-ECD and the variants.***A*, size-exclusion chromatography. The elution volumes of the protein standards are indicated by *arrows*, from which the apparent molecular weights of the purified proteins were estimated. *B*, dynamic light scattering of the wild-type protein and the Q299H variant. Data are expressed as average ± standard deviation (n = 2–3). ∗*p* < 0.05.

In size-exclusion chromatography, all the variants were eluted as a single peak with apparent molecular weights at or greater than that of a homodimer (66 kDa), except that the Q299H variant has a smaller apparent molecular weight at approximately 50 kDa (Fig. 5). Consistently, the hydrodynamic diameter of the Q299H variant (7.4 ± 1.2 nm), which was determined by dynamic light scattering, is significantly smaller than that of the wild-type protein (10.6 ± 0.3 nm, *p* < 0.05). As only one species was detected in both the size-exclusion chromatography and the dynamic light scatter experiment, the monomer–dimer equilibrium appears to strongly favor one side, but the current data cannot tell whether this variant preserves dimerization or becomes monomeric in solution.

**Figure 6:**
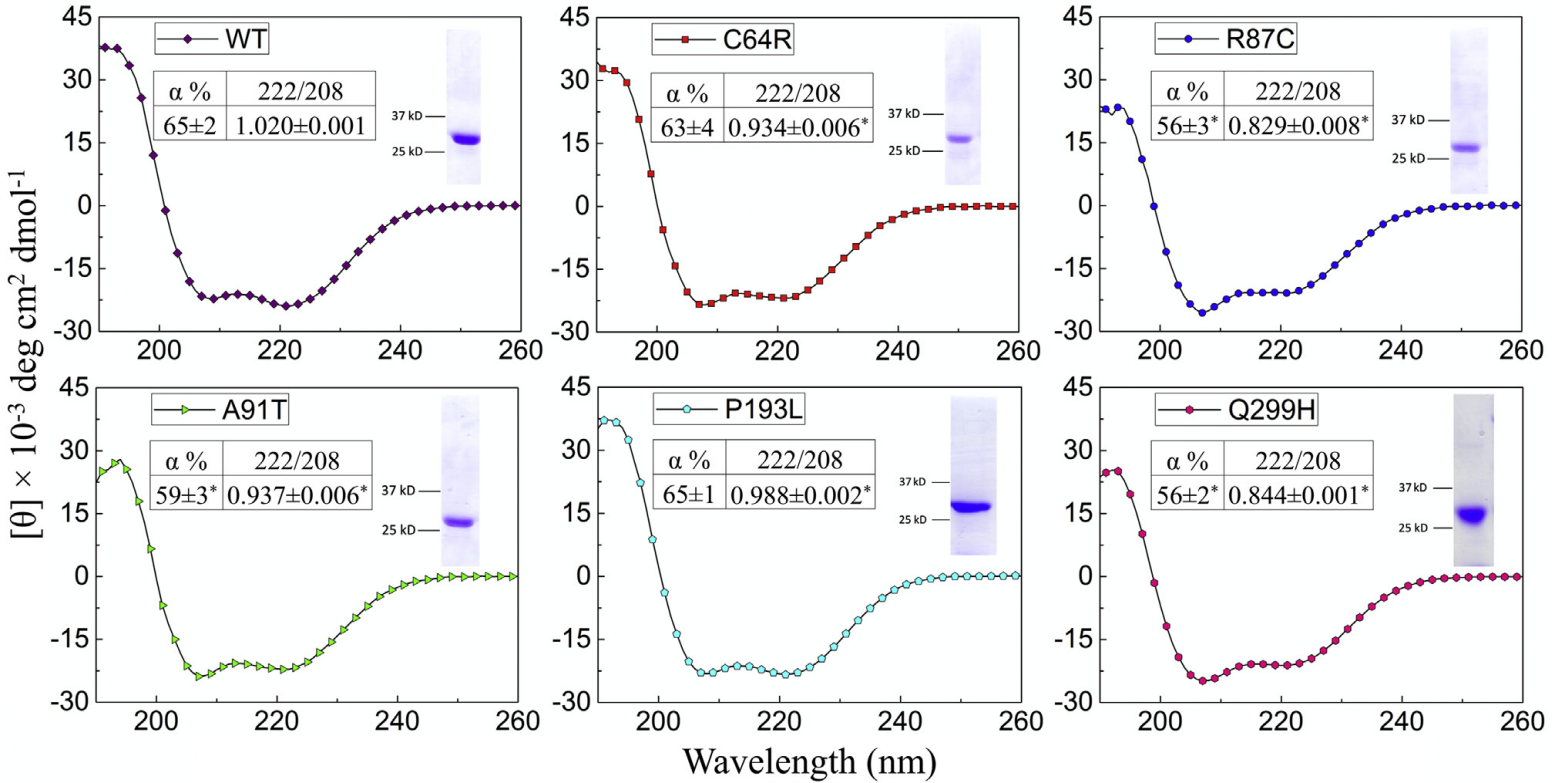
**CD spectra of pZIP4-ECD and the variants.** For each protein, representative spectrum out of three repeats is shown. The α-helical content (α %) was estimated using the K2D3 server. 222/208 indicates the ratio of θ_222_/θ_208_. The SDS-PAGEs of purified protein are shown in the *insets*. The results of 3–5 technical repeats from one experiment are expressed as average ± standard deviation. For the wild-type pZIP4-ECD and the P193L variant, three independent batches of purified proteins were analyzed, from which the α-helical contents were determined to be 66 ± 0% and 65 ± 1%, and the 222/208 ratios are 1.031 ± 0.009 and 0.976 ± 0.016, respectively. ∗*p* < 0.05.

The purified proteins were applied to circular dichroism (CD) spectrometer, and the data were analyzed on the K2D3 server to estimate secondary structure contents (34). As shown in Figure 6, the CD spectrum of the wild-type pZIP4-ECD has a high α-helical content (66 ± 2%) with the characteristic minima at 208 nm and 222 nm and maximum at 195 nm, which is consistent with the reported crystal structure (19). When compared with the wild-type protein, three variants exhibited lower levels of α-helical content – R87C (56 ± 3%), A91T (59 ± 3%), and Q299H (56 ± 2%) (*p* < 0.05). In addition, the ratios of ellipticities (θ) at 222 nm and 208 nm were significantly reduced. The θ_222_/θ_208_ ratio is an indicator of whether α-helices are packed to form coiled-coil-like tertiary structures (35, 36). A value smaller than 1 may indicate poorly packed helices and lack of coiled-coils. For the wild-type protein, the θ_222_/θ_208_ ratio (1.020) is slightly greater than 1, which is consistent with the crystal structure where two helix bundles form two separate subdomains: in the N-terminal helix-rich domain (HRD), α4 is surrounded and packed with the other eight helices, whereas four helices (α10-α12) in the C-terminal domain (PAL motif-containing domain, PCD) are packed against the counterparts from the other monomer to form a homodimer (19). For four variants, the θ_222_/θ_208_ ratios are significantly smaller than 1—C64R (0.934), R87C (0.829), A91T (0.937), and Q299H (0.844) (*p* < 0.05). In contrast, the θ_222_/θ_208_ ratio of the P193L variant (0.998) is only slightly reduced. Together with the unchanged α-helical content, this result shows that this variant, unlike the other variants, has no detectable defects in secondary structure.

**Figure 7:**
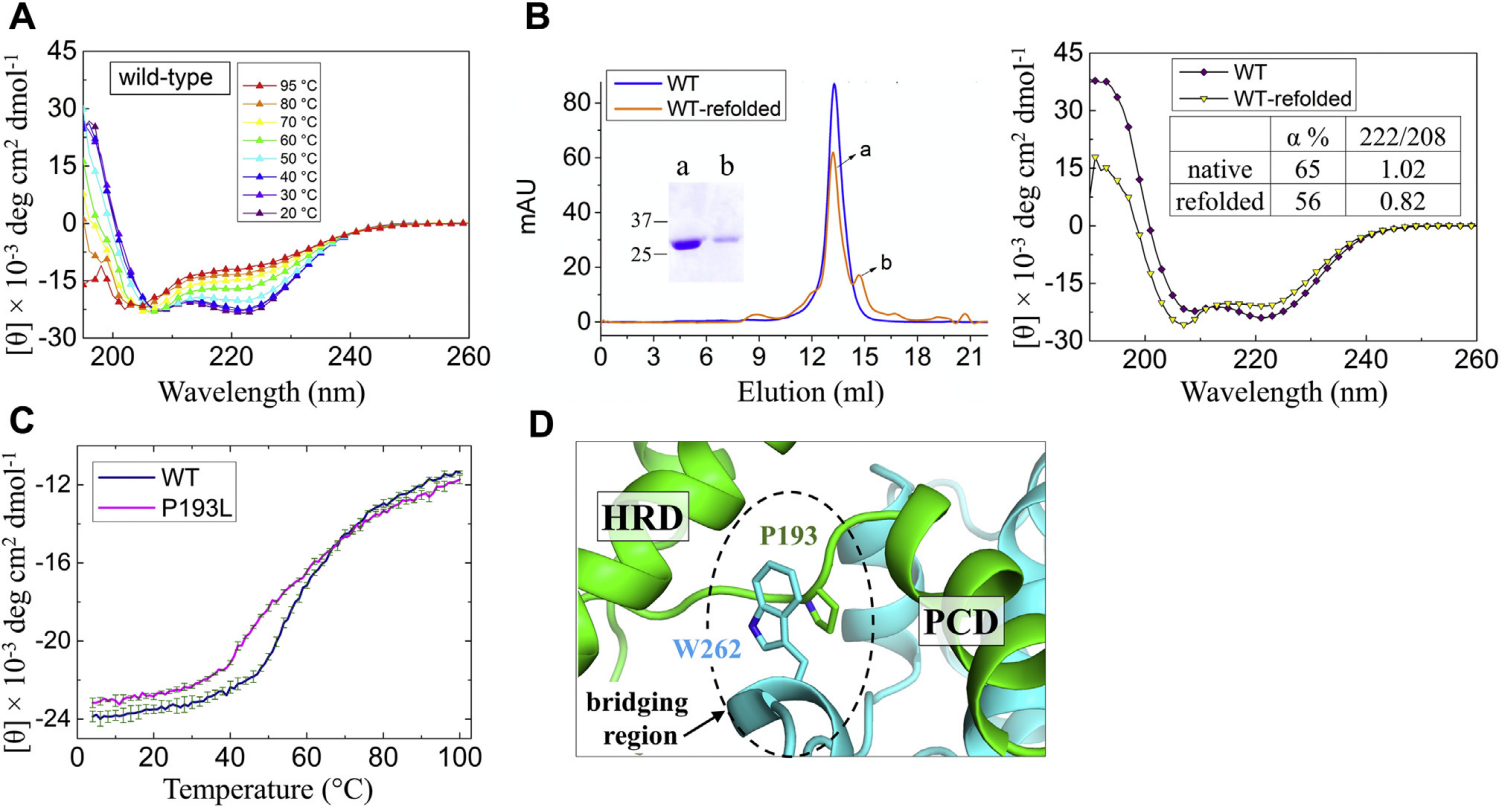
**Thermal stability of the wild-type pZIP4-ECD and the P193L variant.***A*, CD spectra of wild-type pZIP4-ECD at different temperatures. At 95 °C, the α-helical content was estimated to be 20%. *B*, comparison of the native and the refolded pZIP4-ECD. *Left*: size-exclusion chromatography. Peaks a and b are dimer and monomer, respectively, of which SDS-PAGE is shown in the inset. *Right*: CD spectra with indicated α-helix contents and θ_222_/θ_208_ ratios. *C*, heat denaturation curves of the wild-type pZIP4-ECD and the P193L variant. The error bars indicate standard deviations of three technical repeats in one experiment. The shifted curve of the P193L variant relative to the wild-type protein was consistently observed in three independent experiments. *D*, the interaction of P193 with W262 (PDB ID: 4X82). The two monomers are colored in *green* and *cyan*, respectively. The bridging region is indicated by the *dashed oval*.

Next, we examined the thermal stability of the P193L variant and compared it with the wild-type protein. For the latter, increasing temperature from 4 °C to 100 °C at the rate of 0.5 °C/min was accompanied with a decrease of θ_222_ and the plateau was reached at the end when the protein was mostly denatured with α-helical content dropping from 66% to 20% (Fig. 7*A*). Although the ECD has two structurally distinct subdomains (the HRD and the PCD) with the PCD dimerizing through a large hydrophobic interface, the presence of only one transition in the heat denaturation curve suggests that the physical interactions between the two subdomains may synchronize their unfolding processes. However, as pZIP4-ECD could not be fully denatured even at 100 °C, the current data do not allow curve fitting using cooperative unfolding model. We also noticed that, when the heat-denatured protein was allowed to refold at room temperature, the majority of the refolded protein was still a homodimer in solution as indicated in size-exclusion chromatography (Fig. 7*B*), although the α-helical content was decreased by 10% and the θ_222_/θ_208_ ratio was reduced to 0.82. As pZIP4-ECD forms a stable homodimer, heat denaturation may go through two distinct pathways. In the first scenario, while the protein is denatured by heat, the dimer breaks into monomers, most of which return to the dimeric state upon refolded. In the second scenario, dimerization is preserved throughout the process of heat denaturation even when the protein is largely, but not fully, denatured at 100 °C. Although the current data cannot distinguish these pathways, the result already indicates that pZIP4-ECD has a strong propensity to keep or form a dimer, which is consistent with the proposed ECD function of facilitating ZIP4 dimerization (19). When the heat denaturation curve of the P193L variant was compared with that of the wild-type protein, a substantial left shift was observed (Fig. 7*C*), particularly at the first half of the curve, indicating that the helical content of the variant began to decrease at lower temperatures and therefore the thermal stability of this variant is reduced. In the crystal structure of pZIP4-ECD, P193 stacks with W262 from the other monomer through a proline-aromatic interaction (Fig. 7*D*). Alanine substitution of W266 in hZIP4, which is topologically equivalent to W262 of pZIP4-ECD, causes the same immature glycosylation and loss of zinc transport activity as the P200L mutation (19), indicating that this proline–tryptophan interaction is essential for proper trafficking of hZIP4. Reduced thermal stability caused by the proline mutation, as shown in Figure 7*C*, likely accounts for these defects. Although P193 does not directly participate in the hydrophobic cores of either subdomain, the P193L mutation may affect stability of pZIP4-ECD by disrupting domain interactions. As the P193-W262 interaction is located at the bridging region, which mediates communication of the HRD and the PCD, the P193L mutation may negatively affect the cooperativity of the two subdomains during heat denaturation and as such reduce the overall thermal stability. Another possibility, which is compatible with the first theory, is that the P193L mutation may destabilize dimerization since the P193-W262 interaction occurs between the two monomers (Fig. 7*D*). In either scenario (or a combination), disrupted domain interactions would reduce structural compactness and expose hydrophobic patches, making this variant retained in the ER by the quality control system.

Taken together, the *in vitro* studies of purified proteins showed that: (1) most of the variants preserve the oligomeric state, whereas the Q299H variant has a significantly smaller size than a wild-type dimer; (2) three variants (R87C, A91T, and Q299H) exhibit lower α-helical contents; (3) the θ_222_/θ_208_ ratios of four variants (C64R, R87C, A91T, and Q299H) are significantly reduced and lower than one; and (4) the P193L variant exhibits reduced thermal stability. In sum, the substitutions in pZIP4-ECD, which are equivalent to the AE-causing mutations in hZIP4-ECD, lead to structural defects or decrease of thermal stability.

## Discussion

As the ZIP family plays a central role in transition metal homeostasis and cell signaling (18), LOF mutations of human ZIPs cause severe syndromes, including AE, which is the first genetic disorder discovered to be associated with the ZIP family (5, 6). Some of the AE-causing mutations have been biochemically studied in mZIP4, a close homolog of hZIP4. Most of the missense mutations in the TMD of mZIP4 led to lower expression level, reduced cell surface expression, and largely diminished zinc transport activity (7). Mapping the mutations on the structural model of hZIP4 suggests that the involved residues appear to play key structural roles and as such the AE-causing mutations in the TMD would affect protein folding and/or stability (37), leading to degradation by quality control mechanism in the ER (31). The only characterized mutation in the ECD of mZIP4 is P200L, which is equivalent to P200L in hZIP4 (7). The same mutation in hZIP4 was also reported in a recent study (23). In this work, we characterized all the seven confirmed AE-causing mutations occurring in the ECD and found that they exhibited similar behavior when expressed in HEK293T cells—they were able to be expressed but barely detected at cell surface, which accounts for the total loss of zinc transport activity. The fact that the variants are immaturely glycosylated and aberrantly trapped in the ER strongly indicates intracellular mistrafficking. By biophysically studying the corresponding mutations in pZIP4-ECD, we demonstrated that the mutations led to structural defects or decreased thermal stability, which may account for mistrafficking and dysfunction of the AE-associated hZIP4 variants.

Protein misfolding and structural defects are known pathogenic mechanisms for dysfunction of membrane proteins caused by disease-associated mutations. A well-documented case is the autosomal recessive disorder cystic fibrosis (CF), which is caused by LOF mutations of a chloride ion channel, cystic fibrosis transmembrane conductance regulator (CFTR). Notably, the most prevalent mutation (>90%) associated with CFTR is ΔF508, for which the residue F508 within the cytosolic nucleotide binding domain 1 (NBD1) is deleted (38). The ΔF508 variant exhibited drastically reduced transport activity, immature glycosylation, reduced cell surface expression, and accelerated clearance from plasma membrane (39). The atomic resolution of cryo-EM structures of CFTR has shown that F508 is buried in a hydrophobic core (40, 41), which mediates physical interactions of the NBD1 and the TMD, and biochemical/biophysical studies have indicated that the variant has defects in folding and structural compactness (39). Remarkably, the AE-associated variants with single residue substituted in the ECD showed a similar behavior as the ΔF508 CFTR variant. As discussed in our previous report and also shown in Figure 1, the AE-causing mutations occur at the conserved residues in structured regions where they participate in disulfide bonds (C62R, C309Y), salt bridge (R95C), hydrogen bonds (N106K and Q303H), hydrophobic core (A99T), and interdomain interactions (P200L). With no surprise, the biophysical studies on pZIP4-ECD indicated that the tested five variants (C64R, R87C, A91T, P193L, and Q299H) have structural defects (lower α-helical content and reduced θ_222_/θ_208_ ratio) or decreased thermal stability (Figs. 6 and 7). The extremely low yield for the D98K and C305Y variants already implies defects in folding and/or stability. There is some uncertainty whether the mutations would affect the full-length protein in a similar manner as they do on the isolated ECD or whether the observed defects are severe enough to cause mistrafficking. Given that the ECD is a functionally important accessory domain, which may form extensive interactions with the TMD, it is possible that the seemingly modest structural changes may have profound effects on the full-length protein to induce mistrafficking.

We notice the discrepancy between our results and other reports on the P200L variant. It was shown that the P200L variant of mZIP4 was expressed in HEK293T cells with a cell surface level comparable with that of the wild-type protein and a 70% decrease in zinc transport activity (7). In contrast, the P200L variant of hZIP4 showed little surface expression and no detectable activity in this work (Figs. 2 and 3). As already mentioned in the mZIP4 study, nearly all the wild-type mZIP4 was maturely glycosylated, whereas only 50%–70% of the P200L variant did so, suggesting that a significant amount of the variant was not properly processed. Notably, even for the wild-type hZIP4, maturely glycosylated protein is no more than 50% (Fig. 2). It is unclear why mZIP4 is more efficiently processed and traffic in a human cell line than hZIP4. One possibility would be that mZIP4 is intrinsically more stable than hZIP4 so that it has a better chance to fold correctly in the ER. It would also explain why the P200L variant of mZIP4 can still be presented at cell surface despite the folding defect evidenced by immature glycosylation. While this work was being conducted, we noticed a study reporting that the P200L variant of hZIP4 expressed in HEK293T cells was shown to have a normal zinc transport activity, which was measured by using a small-molecule zinc-responsive fluorescence dye (23). It is unclear whether the discrepancy is due to different approaches used for tracing zinc transport. Radioactive ^65^Zn-based transport assay has been used to study transport kinetics of a variety of ZIPs, including hZIP4 (7, 19, 28, 29, 32, 37, 42, 43, 44, 45). Consistent with loss of zinc transport activity, the P200L variant of hZIP4 is shown to be barely expressed at cell surface. We detected cell surface expression levels of hZIP4 and its variants using two approaches following the previous mZIP4 study (7)—western blot for detecting cell surface bound anti-HA antibody and immunofluorescence imaging for visualizing fluorescence-labeled anti-HA antibody associated at cell surface. As shown in Figure 3, *A* and *B*, the results of both approaches consistently showed that the AE-associated variants, including the P200L variant, had much lower levels of cell surface expression than the wild-type hZIP4. Colocalization experiment further revealed that the P200L variant is largely retained in the ER (Fig. 4), which explains the lack of zinc transport activity of this variant.

After the discovery of the causal link between ZIP4 mutations and AE, disease-causing mutations have been reported in other human ZIPs. The LOF mutations of ZIP13 cause the spondylocheirodysplastic form of Ehlers–Danlos syndrome (SCD-EDS) (44, 46, 47), an autosomal recessive disorder characteristic of defects of connective tissues, bones, and teeth. It has been demonstrated that both the missense mutation G64D and the deletion mutation ΔFLA (on the third transmembrane helix) drastically reduced stability and protein expression level in cells due to accelerated clearance through the ubiquitination-dependent proteasome degradation pathway (46). Recently, inherited LOF mutations of ZIP8 and ZIP14, which are close homologs with broad substrate spectrum covering from the beneficial trace elements (zinc, iron, manganese) to the toxic cadmium, have been linked with severe failure of systemic manganese homeostasis (48, 49). The disease-linked ZIP8 variants fail to transport Mn^2+^ because of abrogated cell surface expression and being aberrantly retained in the ER (50). Notably, these ZIP8 variants contain at least one substitution in the ECD, highlighting the importance of the ECD in ZIP8 intracellular trafficking. In this work and also in our previous report, we demonstrate that the mutations on the conserved residues in the ECD significantly affected ZIP4 intracellular trafficking (19). Although the exact role of the ECD is still not fully clarified, these studies have suggested that correct folding and structural integrity of the ECD are required for normal intracellular trafficking of the ZIPs. Whether the ECD plays any active roles in folding, processing, or trafficking of mammalian ZIPs would be interesting to investigate later. Given that AE and CF share a similar molecular pathogenic mechanism and that small-molecule chemical chaperones have been successfully applied for CF treatment (51), developing AE-specific chemical chaperones may represent an alternative strategy to treat this rare disease. After all, lifetime and high-dose zinc administration may cause imbalance of other trace elements, such as copper deficiency (52, 53).

## Experimental procedures

### Genes, plasmids, and mutagenesis

The plasmids harboring hZIP4 (GenBank code: BC062625) or pZIP4-ECD (gene ID: ELK11751, residue 36-322) are the same as reported (19). All the site-directed mutations were conducted using QuikChange mutagenesis kit (Agilent, Cat# 600250) and verified by DNA sequencing.

### Cell culture, transfection, and western blot

Human embryonic kidney cells (HEK293T, ATCC, Cat #CRL-3216) were cultured in Dulbecco’s modified eagle medium (DMEM, Thermo Fisher Scientific, Invitrogen, Cat#11965092) supplemented with 10 % (v/v) fetal bovine serum (FBS, Thermo Fisher Scientific, Invitrogen, Cat#10082147) and Antibiotic-Antimycotic solution (Thermo Fisher Scientific, Invitrogen, Cat# 15240062) at 5% CO_2_ and 37 °C. Cells were seeded on poly-D-lysine (Corning, Cat# 354210) coated 24-well trays for 16 h in the basal medium and transfected with 0.5–0.8 μg DNA/well by lipofectamine 2000 (Thermo Fisher Scientific, Invitrogen, Cat# 11668019) in OPTI-MEM medium.

For western blot, all the samples were heated at 96 °C for 6 min before loading on SDS-PAGE gel. The protein bands were transferred to PVDF membranes (Millipore, Cat# PVH00010). After being blocked with 5% nonfat dry milk, the membranes were incubated with anti-HA antibody (Thermo Fisher Scientific, Cat#26183) at 4 °C overnight. Bound primary antibodies were detected with HRP-conjugated goat anti-mouse immunoglobulin-G at 1:5000 (Cell Signaling Technoloy, Cat# 7076S) or goat anti-rabbit immunoglobulin-G at 1:2500 (Cell Signaling Technology, Cat# 7074S) by chemiluminescence (VWR, Cat# RPN2232). The images of the blots were taken using a Bio-Rad ChemiDoc Imaging System.

### PNGase F and Endo H glycosidase digestion

For PNGase F digestion, 500 unit of enzyme was added to the cells lysed in the SDS-PAGE sample loading buffer and incubated for 1 h at 37 °C before detection using SDS-PAGE and western blot.

For Endo H digestion, cell pellets were dissolved in diluted denaturation solution provided by the Endoglycosidase H kit (Promega). After vigorously mixing and heating at 95 °C for 5 min, the samples were allowed to cool down to room temperature, followed by addition of 10x Endo H reaction buffer and 1250 units of Endo H enzyme and incubation for 15 h at 37 °C. Control samples were treated in the exact same way except that the enzyme was not added. Samples were analyzed using 8% SDS-PAGE and western blot.

### Zinc transport assay

Twenty hours posttransfection, cells were washed with the wash buffer (10 mM HEPES, 142 mM NaCl, 5 mM KCl, 10 mM glucose, pH 7.3) followed by incubation with 10 μM ZnCl_2_ (containing 30% ^65^ZnCl_2_) in Chelex-treated 10% FBS/DMEM media for 20 min at 37 °C. Then, plates were transferred on ice and zinc uptake was stopped by addition of precooled 1 mM EDTA containing wash buffer. Cells were centrifuged at 3500 rpm and the supernatant was discarded. Cells were washed twice before lysis with 0.5% Triton X-100. Packard Cobra Auto-Gamma counter was used to detect radioactivity. The same procedure was used for activity assay of the P200L variant at designated zinc concentrations. The cells transfected with the empty vector were treated in the same way.

### hZIP4-HA surface expression detection

hZIP4-HA expressed at cell surface was indicated by the surface bound anti-HA antibodies recognizing the C-terminal HA tag of hZIP4, as previously described (7, 19, 32, 33). In brief, after 24 h transfection, cells were washed twice with DPBS on ice and then fixed for 10 min in 4% formaldehyde at room temperature. Cells were then washed three times in DPBS (5 min each wash) and incubated with 2.5 μg/ml anti-HA antibody diluted with 5% BSA in DPBS for 1.5 h at room temperature. Cells were washed seven times with DPBS to remove unbound antibodies and then lysed in SDS-PAGE loading buffer. The anti-HA antibody bound to the surface hZIP4-HA in cell lysates was detected in western blot with HRP-conjugated goat anti-mouse immunoglobulin-G (1:5000). As loading control, β-actin levels were detected using an anti-β-actin antibody (1:2500).

### Immunofluorescence imaging and colocalization analysis

HEK293T cells were grown in 24-well trays for 16 h on sterile glass coverslips and transfected with plasmids harboring the genes of hZIP4 or its variants using lipofectamine 2000. To visualize cell surface expressed hZIP4 or its variants, cells were washed briefly by DPBS after 24 h transfection and then fixed for 10 min at room temperature using 4% formaldehyde. The cells were washed in DPBS (5 min each wash) and incubated with 2.5 μg/ml an FITC-labeled anti-HA antibody (Sigma, Product # H7411) diluted with 5% BSA in DPBS for 1.5 h at room temperature. After five washes with DPBS, coverslips were mounted on slides with fluoroshield mounting medium with DAPI (Abcam, Cat# ab104139). Images were taken with a 40X objective using a Spectral-based Olympus FluoView 1000 CLSM.

To detect intracellular localization of hZIP4 variants and calreticulin (an ER marker), after fixation by using formaldehyde, cells were permeabilized and blocked for 1 h with DPBS containing 5% goat serum (Cell Signaling Technology, Cat# 5425S) and 0.1% Triton X-100 and then incubated with anti-HA antibodies at 1:500 (Thermo Fisher Scientific, Cat#26183) and/or anti-calreticulin antibodies at 1:300 (Thermo Fisher Scientific, Cat#PA3-900) at 4 °C for overnight. After three washes with DPBS (5-min incubation for each wash), cells were incubated with Alexa-568 goat anti-mouse antibodies at 1:500 (Thermo Fisher Scientific, Cat# A-11004) and Alexa-488 anti-rabbit antibodies at 1:500 (Thermo Fisher Scientific, Cat# A-27034). After three washes with DPBS, coverslips were mounted on slides with fluoroshield mounting medium with DAPI (Abcam, Cat# ab104139).

For colocalization analysis, dual-channel images (red for hZIP4 and green for calretuculin) were taken with a 100× oil objective. Pearson’s correlation coefficient was calculated to quantify colocalization using Image J equipped with the JACoP plugins (54). PCC reflects the degree of linear correlation between the fluorescence intensities of the colors in a dual-channel image (55). A PCC of +1 indicates a perfect positive correlation, whereas a PCC of −1 indicates the most negative correlation. A PCC of 0 means no correlation. For each construct, 7–10 images were taken and 4–7 images with the best quality were used in statistical analysis.

### Expression and purification of pZIP4-ECD and the variants

The wild-type pZIP4–ECD and the variants were expressed as previously described (19). In brief, the *Escherichia coli* cells of Origami B(DE3) pLysS (Novagen) transformed with the pLW01 vector were grown at 37 °C in lysogeny broth medium until OD_600_ ∼ 0.6, and expression was induced by 0.2 mM IPTG before the cells were transferred to 16 °C for overnight growth. After cell lysis by sonication, the His-tagged proteins were purified using a nickel-nitrilotriacetic acid column. After removal of N-terminal His_6_-tag by thrombin, the proteins were dialyzed against a Tris-HCl buffer (pH 8.0) containing 10 mM EDTA, subjected to ion-exchange chromatography (Mono-Q, GE Healthcare), and then polished by size-exclusion chromatography (Superdex 200 10/300GL, GE Healthcare) in a buffer containing 10 mM HEPES, pH 7.3, and 100 mM NaCl. The apparent molecular weight was estimated using the elution volumes of the protein standards.

### Circular dichroism experiments

To prepare the samples for CD experiments, Thermo Scientific Slide-A-Lyzer MINI Dialysis Device was used for changing the sample buffer to 10 mM phosphate pH 7.3 over the course of a 2-day dialysis at 4 °C with buffer change for three times. Sample concentrations were measured by using a NanoDrop ND-1000 Spectrophotometer and then adjusted to proper concentration using the dialysis buffer. The CD spectrum of 8 μM pZIP4–ECD (or the variants) in 300 μl of 10 mM phosphate buffer (pH 7.3) was recorded in Hellma 1 mm QS cuvette sealed with PTFE stopper by using a JASCO-815 CD Spectrometer. Wavelength range was set between 190 to 260 nm, and data was collected at 1 nm wavelength increments with 1.5 s averaging time per wavelength point. Three scans were recorded and averaged. The K2D3 server (http://cbdm-01.zdv.uni-mainz.de/∼andrade/k2d3/n) was used to estimate secondary structure components of the proteins.

To monitor heat denaturation of the wild-type protein, CD spectra were recorded in the range of 195–260 nm and starting temperature was set at 20 °C. Temperature was increased in 10 °C increments and waiting time was set to 5 min at each designated temperature before next scanning. Three scans were recorded and averaged. After reaching 95 °C, temperature was cooled down to 20 °C and waited for 10 min before scanning. Three scans were recorded and averaged.

For thermal stability study, ellipticities at 222 nm were recorded while the temperature increased from 4 °C to 100 °C. Rate of temperature increase was optimized to 0.5 °C/min with a temperature tolerance of 0.15 °C. The average of three repeats was used in data processing and analysis.

### Dynamic light scattering experiment

Stock solutions of protein samples (WT and the Q299H variant) were dialyzed using Thermo Scientific Slide-A-Lyzer MINI Dialysis Device for changing the buffer to 10 mM phosphate pH 7.3. Concentrations of both protein samples were adjusted to 15 μM and were filtered through 0.22 μm filter to remove large aggregates/dust. DLS was performed using Zetasizer Nano (Malvern) and offset was kept the same for the buffer and protein samples. Each measurement was an average of 20 acquisitions with 12 s per acquisition and performed at 25 °C using 633 nm wavelength.

## Data availability

All data are contained within the article.

## Conflict of interest

The authors declare no conflicts of interest with the contents of this article.
