# Supplementary material for: Zinc transporter mutations linked to acrodermatitis enteropathica disrupt function and cause mistrafficking
Source: J Biol Chem. 2021 Jan 8;296:100269. doi: 10.1016/j.jbc.2021.100269 (PMC7949036; doi:10.1016/j.jbc.2021.100269)
Supplement: Figure S1 [file mmc1.docx]

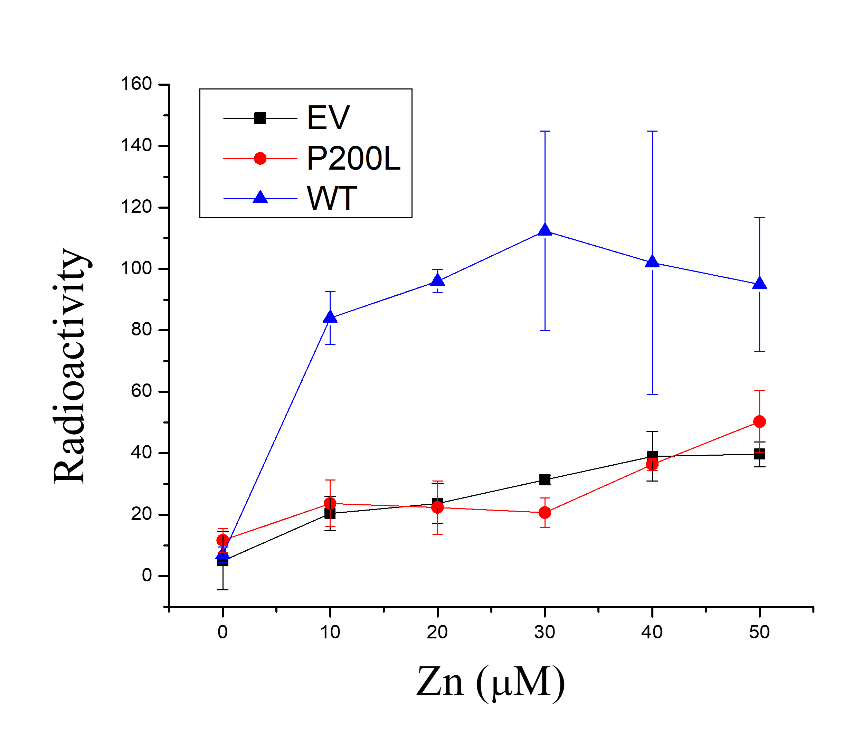
**Figure S1.** Zinc transport activity measurement at indicated zinc concentrations. Three technical repeats were averaged at each data point. The error bars indicate standard deviation (n=3).
